# A comparison of the predictive validity of the combination of the neutrophil-to-lymphocyte ratio and platelet-to-lymphocyte ratio and other risk scoring systems for intravenous immunoglobulin (ivig)-resistance in Kawasaki disease

**DOI:** 10.1371/journal.pone.0176957

**Published:** 2017-05-23

**Authors:** Seiichiro Takeshita, Takashi Kanai, Yoichi Kawamura, Yusuke Yoshida, Shigeaki Nonoyama

**Affiliations:** 1 Division of Nursing, National Defense Medical College, Tokorozawa, Saitama, Japan; 2 Department of Pediatrics, National Defense Medical College, Tokorozawa, Saitama, Japan; 3 Department of Pediatrics, Japan Self-Defense Forces Central Hospital, Setagaya, Tokyo, Japan; Institut National de la Santeet de la Recherche Medicale (INSERM), FRANCE

## Abstract

**Background:**

We recently reported that the combination of the neutrophil-to-lymphocyte ratio (NLR) and the platelet-to-lymphocyte ratio (PLR) is a novel and useful predictor of intravenous immunoglobulin (IVIG)-resistance in Kawasaki disease (KD). In the present study, to evaluate the effectiveness of the new risk score, we compared its predictive validity to that of previously reported risk scores.

**Materials and methods:**

The laboratory records of 437 patients with KD before IVIG therapy were retrospectively analyzed, and the IVIG-responsive (n = 344) and IVIG-resistant (n = 93) patients were compared. The validity of the new score (the combination of NLR≥3.83 and PLR≥150) for predicting IVIG resistance in KD was compared to that of the Kobayashi, Egami and Sano risk scores.

**Results:**

The new score and the Kobayashi score displayed high sensitivity (0.72 and 0.70 respectively) and specificity (0.67 and 0.68 respectively), while the Egami and Sano scores showed high specificity (0.71 and 0.81 respectively) but relatively low sensitivity (0.56 and 0.45 respectively). The odds ratios (ORs) for the new score, the Kobayashi score, the Egami score and the Sano score were 5.34 (95% confidence interval [CI] 3.22–8.85), 4.87 (95% CI 2.96–8.01), 3.14 (95% CI 1.96–5.03) and 3.53 (95% CI 2.17–5.77) respectively.

**Conclusions:**

The predictive validity of the combination of NLR≥3.83 and PLR≥150, which is a simple and convenient indicator, was equal to or higher than that of the other risk scores. This suggests that the new score could be a widely available marker for predicting IVIG resistance in KD.

## Introduction

Kawasaki disease (KD) is an acute febrile illness of unknown etiology that mainly occurs in infants and young children [1]. This disease is characterized by an acute type of systemic vasculitis and patients may develop coronary artery lesions (CAL) [1,2]. Although intravenous immunoglobulin (IVIG) is the established treatment for acute KD [2,3], more than 10% of KD patients are resistant to this therapy. IVIG-resistant patients are at higher risk of developing CAL than IVIG-responsive patients [4,5]. It is important to predict the patients who will be resistant to IVIG before starting the initial treatment, because an intensive initial combination treatment with IVIG and other anti-inflammatory therapies, such as ulinastatin [6], steroid [7,8], infliximab [9] and interleukin (IL)-1 antagonists [10,11], may reduce the occurrence of IVIG resistance and/or CAL. Thus, a clinical tool to predict IVIG resistance before the initial therapy could help clinicians to identify high-risk KD patients would facilitate early intervention and thereby allow the incidence of severe complications to be reduced. There are several risk-scoring systems that use usual laboratory data to predict IVIG resistance in KD patients; the Kobayashi [12], Egami [13] and Sano [14] risk scores have been commonly used in Japan. These risk scores are each composed of 3–7 of the following factors: patient age (months at onset), number of days of illness at diagnosis (IVIG administration), proportion of neutrophils, platelet count, serum sodium level, C-reactive protein (CRP), total bilirubin, aspartate aminotransferase (AST) and alanine aminotransferase (AST).

Recently, two blood cell subtype ratios, the neutrophil-to-lymphocyte ratio (NLR) and the platelet-to-lymphocyte ratio (PLR) have been reported to be useful as systemic inflammatory markers and prognostic indicators of adverse cardiovascular events and cancer [15–18]. We recently reported that a high NLR and PLR before IVIG, especially when combined, can be a novel and useful marker for predicting IVIG resistance in KD patients: an NLR of ≥3.83 or a PLR of ≥150 before IVIG was an independent predictor of IVIG resistance [19]. It is noteworthy that the combination of an NLR of ≥3.83 and a PLR of ≥150 was a more effective predictor than either the NLR or PLR alone [19]. In the present study, we compared the predictive validity of the NLR and PLR with the predictive validity of the Kobayashi, Egami and Sano risk scores to evaluate the effectiveness of our new risk score.

## Materials and methods

### Study design

We retrospectively reviewed the clinical and laboratory findings of 437 KD patients who were hospitalized at the National Defense Medical College hospital between April 2005 and September 2016. KD was diagnosed according to the Diagnostic Guidelines for Kawasaki Disease (5th revision) [20]. The first day of illness was defined as the first day of fever. Patients were excluded if the clinical or laboratory evidence suggested incomplete KD or any other disease that is known to mimic KD, such as adenovirus infection, Epstein-Barr virus infection, scarlet fever, or bacterial cervical lymphadenitis. Patients who presented with CAL before the initial treatment began were also excluded from the study. The present study was approved by the institutional review board at the National Defense Medical College. We obtained written informed consent from the parents or guardians of all KD patients. All of the methods used complied with the relevant approved guidelines.

All of the patients were treated with oral aspirin (30 mg/kg/day), IVIG (2 g/kg/day) and intravenous ulinastatin (15000 U/kg in 3 divided doses) [6]. IVIG resistance was defined as a persistent fever lasting >24 h after the completion of IVIG or recrudescent fever associated with KD symptoms after an afebrile period.

Serial blood samples were obtained during the acute febrile phase before the administration of IVIG and 1 day after IVIG. The baseline characteristics and laboratory data were collected, including the data that have previously been used in the determination of risk scores for IVIG resistance [12–14]. In the case of white blood cell count, proportion of neutrophils, total bilirubin, AST, ALT and CRP, if a laboratory test was performed two or more times before the initial IVIG treatment, the highest value was chosen for the analysis; in the case of the platelet count, albumin and sodium, the lowest value was chosen. As the highest values of AST, proportion of neutrophils and CRP and the lowest values of platelet count and sodium were reported to be chosen in the Kobayashi score [12], the same method was also applied to our new score as well as the Egami and Sano scores to compare their predictive validity under equivalent conditions in the present study.

The neutrophil, lymphocyte, and platelet counts were measured using an automated blood cell counter. The NLR and PLR were calculated by dividing the neutrophil and platelet counts by the lymphocyte count respectively. The patients with the combination of NLR≥3.83 and PLR≥150 before initial treatment were defined as having a high risk of IVIG resistance [19]. The Kobayashi [12], Egami [13] and Sano [14] risk scores were also calculated, and the patients with a high risk of IVIG resistance (≥5 points, ≥3 points and ≥2 points respectively) were detected according to the protocols of each risk score.

The sensitivity, specificity, positive predictive value, negative predictive value and diagnostic accuracy of the combination of NLR≥3.83 and PLR≥150, the Kobayashi, Egami and Sano risk scores for predicting IVIG-resistant patients were assessed. The odds ratios (ORs) were calculated for the combination of NLR≥3.83 and PLR≥150, Kobayashi, Egami and Sano risk scores respectively, to compare the magnitude of the prediction of high-risk patients to the actual outcome of IVIG resistance.

Echocardiography was basically performed on admission, before and after IVIG, and then once every 2–4 days until discharge. CAL was diagnosed in accordance with the Japanese Ministry of Health criteria: an internal lumen diameter of >3.0 mm in children of <5 years of age (>4.0 mm in children of ≥5 years of age) or an internal segment with a diameter at least 1.5 times larger than the diameter of the adjacent segment.

### Statistical analyses

All of the data are presented as the median (25th-75th percentiles) for continuous variables or as percentages for categorical variables. The statistical analyses were performed using EZR (Saitama Medical Center, Jichi Medical University, Saitama, Japan), which is a graphical user interface for the R software program (The R Foundation for Statistical Computing, Vienna, Austria). More precisely, it is a modified version of R commander, which was designed to add the statistical functions that are frequently used in biostatistics [21]. The baseline characteristics and laboratory data were compared between the IVIG-responsive and IVIG-resistant groups using the Mann-Whitney U test for continuous variables and Fisher’s exact test for categorical variables. The ORs were calculated as the crude ORs by a univariate analysis and were expressed with 95% confidence intervals (CI). *P* values of <0.05 were considered to indicate statistical significance.

## Results

### The characteristics of the study participants

Out of the 437 patients who were enrolled in the present study, 93 (21.3%) were resistant to the initial IVIG therapy. The comparison of the clinical and laboratory data between the IVIG-responsive and IVIG-resistant groups (Table 1), revealed that the IVIG-resistant group was significantly older (P = 0.002), had fewer days of illness at the diagnosis (P<0.001), had fewer days of illness at the administration of IVIG (P<0.001), and had a higher percentage of CAL (P<0.001) than the IVIG-responsive group. Before IVIG therapy, the IVIG-resistant group had a significantly higher neutrophil count (P<0.001), neutrophil percentage (P<0.001), NLR (P<0.001), PLR (P<0.001), total bilirubin (P<0.001), AST (P<0.001), ALT (P = 0.001), and CRP (P<0.001) values, and significantly lower lymphocyte (P<0.001) and platelet counts (P<0.001) and a lower serum sodium concentration (P<0.001) than the IVIG-responsive group.

**Table 1:** The clinical data of the IVIG-responsive and IVIG-resistant patients with Kawasaki disease.


	IVIG-responsive (n = 344)	IVIG-resistant (n = 93)	P value
Male (%)	197 (57.3%)	53 (57.0%)	1.00*
Age (months at onset)	25 (13–42)	35 (19–56)	0.002
Age ≤ 6 months	38 (11.0%)	9 (9.7%)	0.85*
Age ≤ 12 months	81 (23.5%)	18 (19.4%)	0.49*
Number of days of illness at the diagnosis	5 (4–5)	4 (3–5)	< 0.001
Number of days of illness at IVIG administration	5 (5–6)	4 (4–5)	< 0.001
CAL(+)	1 (0.3%)	10 (10.8%)	< 0.001*
Laroratory data before IVIG			
WBCs, × 10^3^/mm^3^	13.70 (11.50–17.00)	14.10 (11.00–18.00)	0.96
Neutrophils, × 10^3^/mm^3^	9.32 (7.27–12.0)	10.80 (8.80–15.38)	< 0.001
Neutrophils, %	70.0 (59.2–79.3)	83.5 (76.5–88.9)	< 0.001
Lymphocytes, × 10^3^/mm^3^	2.98 (1.79–4.41)	1.44 (1.07–2.33)	< 0.001
Others, × 10^3^/mm^3^	1.04 (0.64–1.43)	0.70 (0.42–1.06)	< 0.001
Platelet count, × 10^4^/mm^3^	33.20 (27.95–39.15)	30.30 (23.10–35.70)	< 0.001
NLR	3.32 (1.93–6.02)	8.05 (4.53–13.77)	< 0.001
PLR	121.20 (85.37–191.24)	218.75 (157.89–288.53)	< 0.001
Total bilirubin, mg/dl	0.6 (0.4–0.8)	1.0 (0.5–2.6)	< 0.001
AST, IU/L	38 (29–73)	65 (31–158)	< 0.001
ALT, IU/L	25 (14–99)	73 (16–256)	0.001
Albumin, g/dL	3.6 (3.3–3.9)	3.6 (3.2–3.9)	0.29
Sodium, mmol/L	135 (133–136)	133 (130–135)	< 0.001
CRP, mg/dL	7.2 (4.7–10.8)	10.8 (6.8–14.8)	< 0.001

ALT, alanine aminotransferase; AST, aspartate aminotransferase; CRP, C-reactive protein; WBC, white blood cell.

The data are presented as the median (25th-75th percentile) for the continuous variables and as the number of patients (%) for the categorical variables.

The P values were obtained using the Mann-Whitney U test or

*Fisher’s exact test.

### The predictive validity of the Kobayashi, Egami and Sano scores and the new risk score for IVIG resistance

The sensitivity, specificity, positive predictive value, negative predictive value, diagnostic accuracy and the OR for predicting IVIG resistance in KD were compared among the Kobayashi score (≥5 points), Egami Score (≥3 points), Sano score (≥2 points) and the new score (the combination of NLR≥3.83 and PLR≥150) (Table 2). The Kobayashi score and the new score had high sensitivity (0.70 and 0.72 respectively) and specificity (0.68 and 0.67 respectively). The Egami and Sano scores had high specificity (0.71 and 0.81 respectively) but relatively low sensitivity (0.56 and 0.45 respectively). The ORs were high in order of the new score (5.34, 95% confidence interval [CI] 3.22–8.85), Kobayashi score (4.87, 95% CI 2.96–8.01), Sano score (3.53, 95% CI 2.17–5.77) and Egami score (3.14, 95% CI 1.96–5.03).

**Table 2:** The OR, sensitivity and specificity of the Kobayashi, Egami and Sano scores and our new scoring system.

	Sensitivity	Specificity	Positive predictive value	Negative predictive value	Diagnostic accuracy	OR (95% CI)	P
Kobayashi score ≥ 5	0.70	0.68	0.37	0.89	0.68	4.87 (2.96–8.01)	<0.001
Egami score ≥ 3	0.56	0.71	0.34	0.86	0.68	3.14 (1.96–5.03)	<0.001
Sano score ≥ 2	0.45	0.81	0.39	0.85	0.74	3.53 (2.17–5.77)	<0.001
NLR ≥ 3.83 and PLR ≥ 150	0.72	0.67	0.37	0.90	0.68	5.34 (3.22–8.85)	<0.001

CI, confidence interval; OR, odds ratio. The parameters of the Kobayashi score [12] are as follows: 1) sodium ≤133 nmol/ml, 2 points; 2) days of illness at initial treatment ≤4 days, 2 points; 3) AST ≥100 IU/L, 2 points; 4) % of neutrophils ≥80, 2 points; 5) CRP ≥10 mg/dl, 1 point; 6) age ≤12 months, 1 point; and 7) platelets ≤300×10^3^/mm^3^, 1 point. The parameters of the Egami score [13] are as follows: 1) ALT ≥80 IU/L, 2 points; 2) days of illness ≤4 days, 1 point; 3) CRP ≥8 mg/dl, 1 point; 4) age ≤6 months, 1 point; and 5) platelet ≤300×10^3^/mm^3^, 1 point. The parameters of the Sano score [14] are as follows: AST ≥200 IU/L, 1 point; 2) CRP ≥7 mg/dl, 1 point; and 3) total bilirubin ≥0.9 mg/dl, 1 point.

## Discussion

In the present study, the new score (the combination of NLR≥3.83 and PLR≥150) had high sensitivity and specificity for predicting IVIG resistance in KD, and the OR was equal to or higher than the ORs of the other risk scores. As the Kobayashi, Egami and Sano scores consist of 7, 5 and 3 factors, respectively (Table 2), clinicians need to calculate the total sum of each of the points. On the other hand, the NLR and PLR can be calculated quickly and easily with the blood cell count data alone, which can even be obtained from emergent blood tests. The greatest advantage is that the NLR and PLR are routinely detectable indicators that can be obtained without additional cost. Thus, the new score may be more convenient and cost-effective than the Kobayashi, Egami and Sano risk scores.

Neutrophils, which are the most abundant type of white blood cell, play an important role in the inflammatory process. An elevated neutrophil count is a classical marker of inflammation. In particular, it is reported that critically ill patients with shock and sepsis have marked neutrophilia and lymphocytopenia, and that the severity of the clinical course is correlated with the divergence of the neutrophil (higher) and lymphocyte (lower) counts [22]. The platelet count can also increase in response to systemic infection and inflammation, because the increased levels of proinflammatory cytokines lead to the proliferation of megakaryocytes [23,24]. An increased platelet count may reflect the activity of the inflammation pathway. In recent years, there has been growing interest in simple hematological parameters such as the NLR and PLR [15–17], which are thought to be useful markers of the severity of the systemic inflammatory response. Furthermore, several reports indicate that the combination of the NLR and PLR is useful for predicting the risk of cardiac events and mortality in patients undergoing percutaneous coronary intervention [16,25] and predicting a poor prognosis in patients with malignant diseases [26,27].

The usefulness of a risk score for predicting IVIG resistance in KD patients is that it allows for the identification of patients who might be expected to receive a benefit from additional primary anti-inflammatory therapies [6,9]. Although the Kobayashi [12], Egami [13] and Sano [14] risk scores, which are calculated using usual laboratory data, have been widely used in Japan, these scoring systems do not precisely predict IVIG resistance in patients in North America [28] and the United Kingdom [29]. The reason for these regional differences could be due to genetic differences or other environmental factors [30]. We recently reported that two simple ratios, the NLR (≥3.83) and PLR (≥150), can independently predict IVIG resistance in KD patients, and that the combination (NLR≥3.83 and PLR≥150) had a much greater predictive ability than either the NLR and PLR alone [19]. In addition, Ha et al. reported that a high NLR (≥1.0) at 2 days after IVIG predicted the development of CAL and IVIG resistance [31], while Demir et al. reported that the NLR values of KD patients with CAL were significantly higher than the NLR values of KD patients without CAL [32]. Thus, there is value in investigating whether the NLR and PLR can be used to predict IVIG resistance and/or CAL, in other countries.

A number of molecular and immunological biomarkers for predicting IVIG resistance in KD have been reported [33]. The circulating levels of inflammatory cytokines (IL-1β, TNF-α, IL-6, G-CSF), polycythemia rubra vera-1 (PRV-1), matrix metalloproteinase-8 (MMP-8) and damage-associated molecular pattern molecules (DAMPs), such as high-mobility group protein B1 (HMBG1), S100AS and S100A9, have been reported to be higher in IVIG-resistant patients than in IVIG-responsive patients with KD [33]. As IL-1, IL-6 and G-CSF can stimulate the proliferation and differentiation of myeloid cells, the increased production of these cytokines may induce neutrophilia during the acute phase of KD. In addition, Kuo et al. reported that the increase in numbers of eosinophils after IVIG therapy had an inverse correlation with the incidence of IVIG resistance in KD [34]. Thus, the balance of neutrophil and eosinophil counts before and after IVIG therapy might be another candidate marker for predicting and judging the IVIG resistance in KD.

The present study is associated with several limitations. First, we investigated a small number of patients in a single institution. Since we treated all of the KD patients with the initial therapy of IVIG and ulinastatin [6], the proportion of IVIG-resistant patients may be slightly different from that among patients who receive other therapies, such as IVIG alone [2] or IVIG plus steroid [7]. Thus, the OR may also vary slightly depending on the initial therapy protocol. We are currently performing a multi-center analysis using both the development and validation dataset in KD patients treated with IVIG alone. Second, since the present study was retrospective in nature and was performed without randomization, it may have involved some bias. However, we enrolled all of the KD patients admitted to the National Defense Medical College hospital between April 2005 and September 2016 in the present study in an effort to reduce the possibility of a selection bias, and blood tests were performed before IVIG therapy in all of them. Thus, our results were unlikely to be explained by selection bias. In addition, 15%-20% of KD patients were reported to be resistant to initial IVIG therapy in a nationwide survey in Japan [3], while the incidence of initial IVIG-resistance was reported to increase between 2003–2014 in a prefecture of Japan [35]. Although 93 (21.3%) out of the 437 KD patients were resistant to the initial IVIG therapy in the present study, the slight difference in the incidence is thought to be explainable by inter-regional discrepancies. Third, since the percentage of KD patients with CAL was small (only 11 patients [2.51%] out of 437 KD patients in total; Table 1), we could not investigate the usefulness of the NLR and PLR in predicting CAL development. However, despite these limitations, the present results may offer new insight into the risk scores for IVIG resistance in KD patients. A larger well-designed, prospective study should be performed to validate the usefulness of the NLR, PLR and the combination of the two ratios in predicting IVIG resistance and CAL development in KD patients.

## Conclusion

The combination of NLR≥3.83 and PLR≥150 had high sensitivity, specificity and a high OR for predicting IVIG resistance in KD, and the predictive validity of the new risk score was equal to or higher than the predictive validity of the Kobayashi, Egami and Sano risk scores. As the NLR and PLR are simpler and more convenient to obtain than the other scores, the new score could be a widely available marker for predicting IVIG resistance in KD.

## Supporting information

S1 Dataset The proportion of IVIG-responsive and resistant patients in the low and high risk groups of the Kobayashi, Egami, Sano scores and our new score. (XLSX)
